# Development of nanoparticles derived from corn as mass producible bionanoparticles with anticancer activity

**DOI:** 10.1038/s41598-021-02241-y

**Published:** 2021-11-24

**Authors:** Daisuke Sasaki, Kosuke Kusamori, Yukiya Takayama, Shoko Itakura, Hiroaki Todo, Makiya Nishikawa

**Affiliations:** 1 Laboratory of Biopharmaceutics, Faculty of Pharmaceutical Sciences, Tokyo University of Science, 2641 Yamazaki, Noda, Chiba 278-8510 Japan; 2 Faculty of Pharmacy and Pharmaceutical Sciences, Josai University, 1-1 Keyakidai, Sakado, Saitama 350-0295 Japan

**Keywords:** Drug delivery, Nanoparticles, Biomaterials

## Abstract

Recent studies showed that plant-derived nanoparticles (NPs) can be easily produced in high yields and have potential applications as therapeutic agents or delivery carriers for bioactive molecules. In this study, we selected corn as it is inexpensive to grow and mass-produced globally. Super sweet corn was homogenized in water to obtain corn juice, which was then centrifuged, filtered through a 0.45-μm-pore size syringe filter, and ultracentrifuged to obtain NPs derived from corn, or corn-derived NPs (cNPs). cNPs obtained were approximately 80 nm in diameter and negatively charged (− 17 mV). cNPs were taken up by various types of cells, including colon26 tumor cells and RAW264.7 macrophage-like cells, with selective reduction of the proliferation of colon26 cells. Moreover, cNPs induced tumor necrosis factor-α release from RAW264.7 cells. cNPs and RAW264.7 in combination significantly suppressed the proliferation of colon26/fluc cells. Daily intratumoral injections of cNPs significantly suppressed the growth of subcutaneous colon26 tumors in mice, with no significant body weight loss. These results indicate excellent anti-tumor activity of cNPs.

## Introduction

Nanoparticles (NPs), including liposomes and lipid emulsions, particularly those having diameter of approximately 100 nm, have been developed and used as delivery carriers for various pharmaceutical agents^1^. The properties and functions of these synthetic NPs can be easily modulated and controlled by altering their size or surface modification with functional molecules^2,3^. However, the preparation and development of these NPs for clinical applications is a complex and expensive process^4,5^. Recently, extracellular vesicles (EVs), such as exosomes and microvesicles, have been recognized as a novel area of research for the development of NPs. They are released from mammalian cells and play important role in intercellular communications by carrying biologically active molecules, such as lipids, mRNAs, and microRNAs^6^. Researchers have attempted to elucidate the functions and therapeutic potential of EVs, and demonstrated the presence of disease-specific EVs and their potential as drug delivery carriers^7,8^. Although the development of EV-based therapeutic systems is highly suggested for disease treatment owing to their excellent function and high biocompatibility, but low productivity of EVs greatly limits their clinical applications^9,10^.

In addition to these studies, some researchers have revealed the existence of NPs in edible plants and isolated them as edible plant-derived NPs (epNPs)^11^. These epNPs have been prepared from various edible plants, such as grapefruit^12^, ginger^13^, grape^14^, apple^15^, and broccoli^16^, and they shared common characteristics, including nanometer size, ease of preparation, and mass production at low cost. Furthermore, epNPs have been reported to contain biologically active molecules, such as polyphenols^13^ and microRNAs^14,17^, and have been used to deliver anti-cancer agents^18–20^ and small interfering RNA^21–24^ after encapsulation. Therefore, epNPs are anticipated as novel therapeutic agents or drug delivery carriers alternative to synthetic NPs or EVs.

Corn (*Zea mays*) is the most widely grown grain crop throughout the world. Corn is one of the most important food crops and one of the top grains produced among the world’s top seven cereals^25^. It provides many vitamins and essential minerals along with fibers^26^, and it is an accessible source of energy due to its low cost and mass production in the world. Therefore, we speculated that NPs derived from corn could solve the problems of high cost and complex process of synthetic NPs and low productivity of EVs. Furthermore, corn contains vitamins, essential minerals, microRNAs, and xanthophylls, such as lutein and zeaxanthin^27–29^. A previous report showed potent anticancer activity of lutein and zeaxanthin^30^. Collectively, besides being cost effective, NPs prepared from corn are likely novel functional and bioactive NPs containing microRNAs and xanthophylls. However, there have been no studies on NPs prepared from corn. Therefore, in the present study, we prepared and evaluated the characteristics, functions, and biological activities of NPs derived from corn, or corn-derived NPs (cNPs). Moreover, we examined the anticancer activity of cNPs in tumor-bearing mice.

## Results

### Preparation and characterization of cNPs

**Figure 1:**
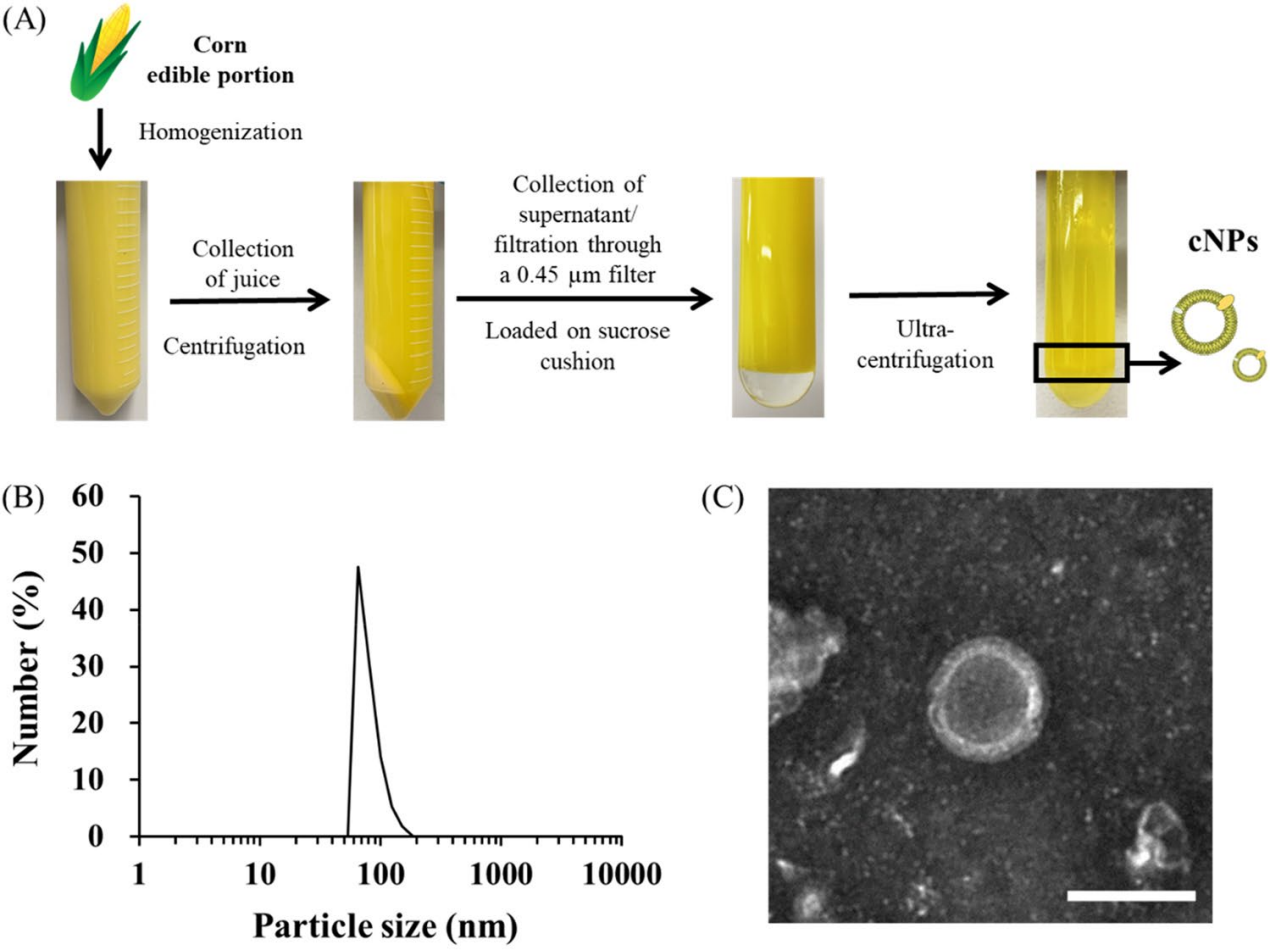
Preparation and characterization of cNPs. **(A)** Flow diagram of cNP preparation. Corn homogenate was obtained from the edible portion of corn using a food processor. The homogenate was centrifuged, filtered, and ultracentrifuged using a sucrose cushion. cNPs were then collected from the indicated phase (black rectangle). **(B)** Size distribution of cNPs was determined using ELSZ-2000ZS. **(C)** TEM image of cNPs was observed using H-7650 TEM. The scale bar indicates 100 nm.

Figure 1A shows the process of preparation of cNPs from homogenized corn juice using sucrose cushion ultracentrifugation method. Dynamic light scattering (DLS) analysis showed that cNPs were uniformly sized with an average particle size of approximately 80 nm and a zeta potential of approximately − 17 mV (Fig. 1B and Table 1). These parameters were almost constant among preparations (independent experiments), indicating that cNPs were generated with high reproducibility. Transmission electron microscopy (TEM) images revealed that cNPs have a hollow structure (Fig. 1C). In addition, the number of cNPs collected from corn was approximately 35.6 ± 1.9 × 10^11^ particles/mL (Table 1), indicating the ease of preparation and mass production of cNPs. Liquid chromatography coupled with tandem mass spectrometry (LC–MS/MS) analysis showed that cNPs contained lysophosphatidylcholines (LPCs), phosphatidylcholines (PCs), lysophosphatidylethanolamines (LPEs), phosphatidylethanolamines (PEs), phosphatidylglycerols (PGs), phosphatidylserines (PSs), and sphingomyelins (SMs) (Table S1).

**Table 1:** Characteristics of cNPs or PC-Lips.

NPs	Particle size (nm)	Zeta potential (mV)	Particle number (× 10^11^ particles/mL)
cNPs	83.3 ± 22.2	− 17.4 ± 1.8	35.6 ± 1.9
PC-Lips	83.6 ± 5.7	5.28 ± 0.2	3.2 ± 1.1

Results are expressed as the mean ± standard deviation (SD) of three independent experiments.

### Cellular uptake of DiI-labeled cNPs

**Figure 2:**
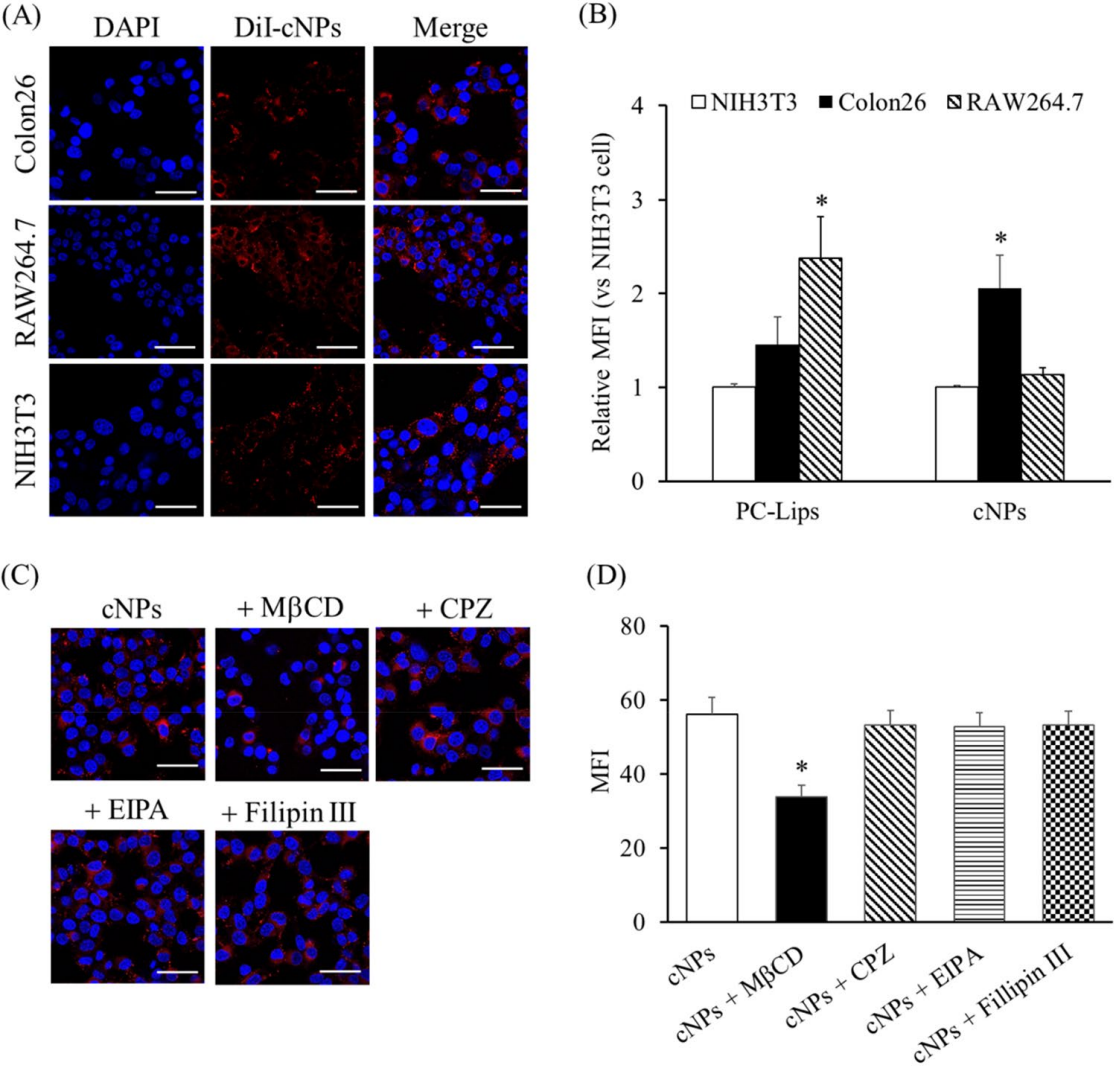
Cellular uptake of cNPs via endocytosis pathway. **(A)** Confocal microscopic images of DiI-labeled cNPs taken up by colon26, NIH3T3, and RAW264.7 cells. Cells were incubated with DiI-labeled cNPs for 3 h. Scale bars indicate 50 μm. **(B)** Quantitative flow cytometric analysis of cellular uptake of DiI-labeled cNPs or PC-Lips. The cells were incubated with DiI-labeled cNPs or PC-Lips for 3 h at 37 °C and fixed using 4% paraformaldehyde phosphate buffer solution (PFA). MFI of cNPs or PC-Lips in the cells was quantified by flow cytometry. Results are expressed as the mean ± SD of three samples. *p < 0.05 vs. NIH3T3 cell group. **(C)** Colon26 cells were incubated with endocytosis inhibitors (0.5 μg/mL Filipin III, 30 μM CPZ, 40 μM EIPA, and 10 mM MβCD) for 40 min and then incubated with DiI-labeled cNPs for 3 h. Cells were fixed and imaged by confocal laser scanning microscopy. Scale bars indicate 50 μm. **(D)** Quantitative flow cytometric analysis of DiI-labeled cNPs taken up by colon26 cells. Endocytosis inhibitors (0.5 μg/mL Filipin III, 30 μM CPZ, 40 μM EIPA, and 10 mM MβCD) were added to colon26 cells and incubated for 40 min. The cells were incubated with DiI-labeled cNPs for 3 h at 37 °C and fixed using 4% PFA. MFI of cNPs in colon26 cells was quantified by flow cytometry. Results are expressed as the mean ± SD of three samples. *p < 0.05 vs. cNP group.

We examined the cellular uptake of cNPs. Figure 2A shows the confocal microscopic images of murine colon adenocarcinoma cell line colon26, murine fibroblast cell line NIH3T3, and murine macrophage-like cell line RAW264.7 cells after addition of 1,1′dioctadecyl-3,3,3′,3′-tetramethylindocarbocyanine perchlorate (DiI)-labeled cNPs. The red fluorescence signals derived from DiI-labeled cNPs were detected in all cell types (Fig. 2A), indicating efficient uptake of cNPs by these cells. To quantitatively evaluate the cellular uptake of cNPs, the mean fluorescence intensity (MFI) of cells after 3 h incubation with DiI-labeled cNPs or PC-liposomes (PC-Lips; control NPs) was calculated. DiI-labeled cNPs were taken up most efficiently by colon26 cells, whereas DiI-labeled PC-Lips were most efficiently taken up by RAW264.7 cells (Fig. 2B). We elucidated the uptake mechanism of cNPs in colon26 cells using endocytosis inhibitors, including methyl-beta-cyclodextrin (MβCD), chlorpromazine (CPZ), ethyl-isopropyl amiloride (EIPA), and Filipin III. MβCD, an inhibitor of cholesterol-dependent endocytosis, significantly inhibited the uptake of DiI-labeled cNPs in colon26 cells; however, CPZ, EIPA, and Filipin III did not. These results suggested involvement of cholesterol-dependent pathway in the uptake of cNPs by colon26 cells (Fig. 2C,D).

### Effect of cNPs on cell number and proliferation

**Figure 3:**
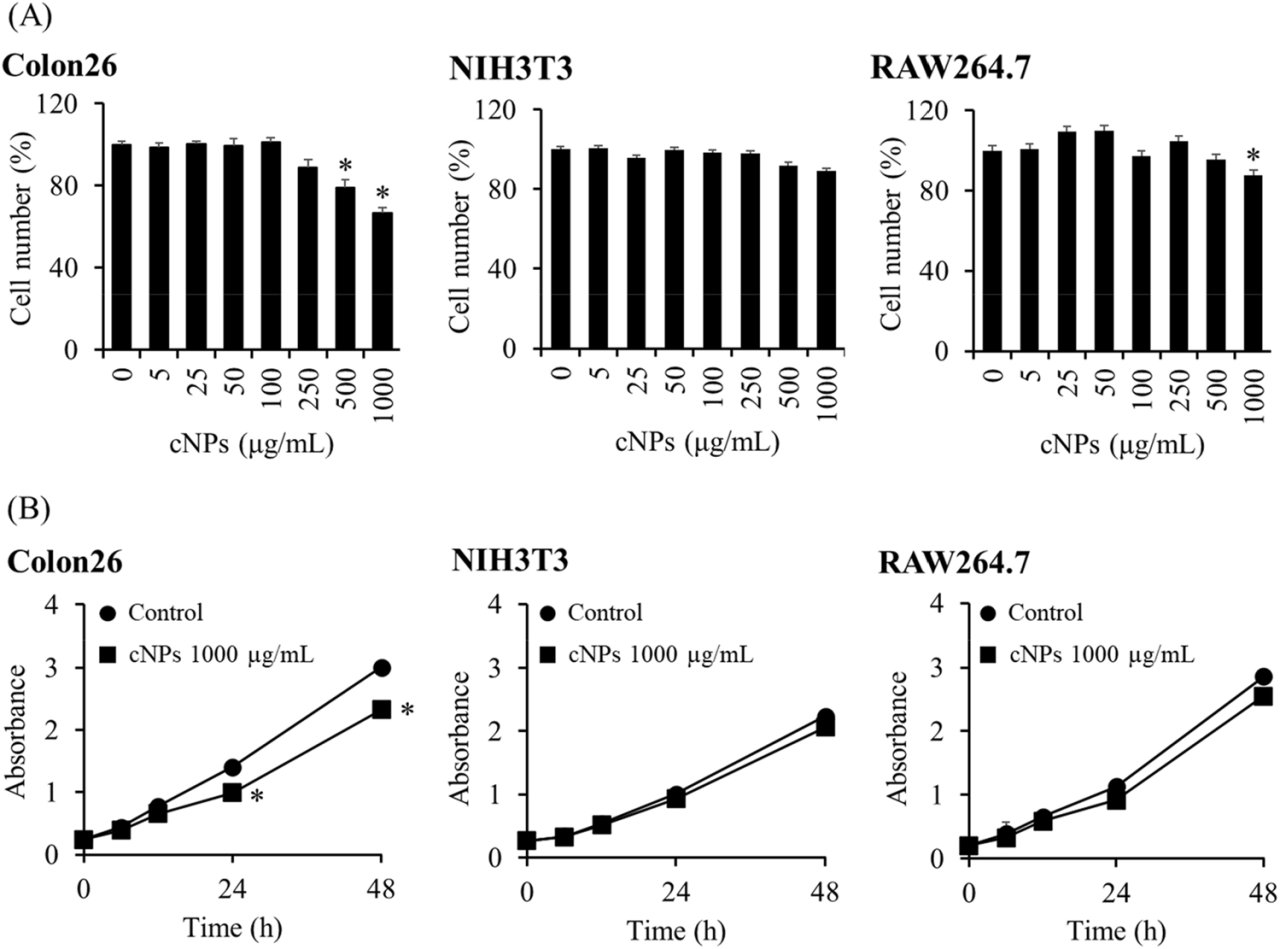
Cell number and proliferation after incubation with cNPs. **(A)** Cell number was measured by Cell Counting Kit-8 (CCK8) assay after 24 h of cNP addition. Colon26, NIH3T3, and RAW264.7 cells were incubated with 5–1000 μg/mL of cNPs. Results are expressed as the mean ± SD of four samples. *p < 0.05 vs. 0 μg/mL cNP group (the control group). **(B)** Cell proliferation was measured by CCK8 assay after 6, 12, 24 and 48 h of 1000 μg/mL cNP addition. Results are expressed as the mean ± SD of four samples. *p < 0.05 vs. control group.

We evaluated the effect of cNPs on the number of colon26, NIH3T3, and RAW264.7 cells after 24 h of incubation. The number of colon26 tumor cells was significantly reduced at a concentration of 500 μg/mL or higher of cNPs compared to normal (NIH3T3) and immune (RAW264.7) cells that lined up at a concentration of 500 μg/mL (Fig. 3A). Figure 3B shows the number of cells over time after incubating them with cNPs at a concentration of 1000 μg/mL; cNPs significantly suppressed the proliferation of colon26 cells compared to that of NIH3T3 and RAW264.7 cells, which were not affected. In addition, we evaluated the effect of cNPs on human cancer cell lines: human breast cancer cell line MDA-MB-231 cells and human pancreatic cell line Panc-1 cells. Consistently, cNPs reduced the number of these human cancer cells (Fig. S2).

### Effect of cNPs on cell cycle and cell death of colon26 tumor cells

**Figure 4:**
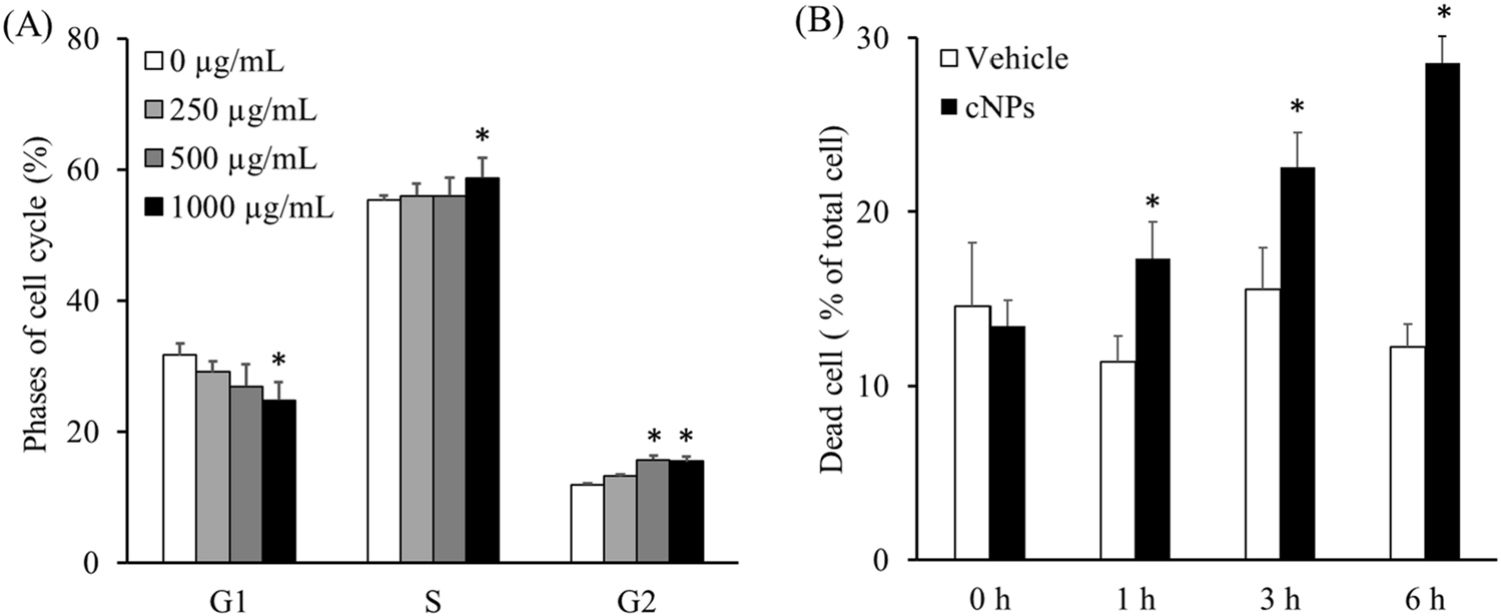
Analysis of cell cycle and dead cells after incubation with cNPs. **(A)** Colon26 cells were incubated with 250, 500, and 1000 µg/mL of cNPs. At 1, 3, and 6 h of incubation, the cells were stained with propidium iodide (PI). Cell cycle analysis was performed using flow cytometry. Results are expressed as the mean ± SD of four samples. *p < 0.05 vs. 0 μg/mL cNP group (control group). **(B)** Colon26 cells were incubated with 1000 µg/mL cNPs or PBS (vehicle). At 1, 3, and 6 h of incubation, the dead cells were stained with PI and detected by flow cytometry. Results are expressed as the mean ± SD of four samples. *p < 0.05 vs. vehicle group.

As the proliferation of colon26 tumor cells was significantly inhibited by cNPs, the effect of cNPs on the cell cycle of colon26 cells was examined. Figure 4A shows the cell cycle phases of colon26 cells with or without 24 h incubation with cNPs at varying concentrations. The proportion of the G1 phase in colon26 cells treated with cNPs decreased in a concentration-dependent manner in contrast to the proportion of the G2 and S phases that increased with cNP treatment. These results suggested that cNPs induced cell cycle arrest at the G2 phase in colon26 cells, leading to a low proportion of the G1 phase and a high proportion of the S/G2 phases.

To elucidate further, colon26 cells were stained with PI at 1, 3, and 6 h after addition of cNPs. Flow cytometry analysis showed that the proportion of dead cells in the vehicle group did not change significantly at any time point, whereas it was increased in the cNP group in a time-dependent manner (Fig. 4B).

### Cytokine release from RAW264.7 cells

**Figure 5:**
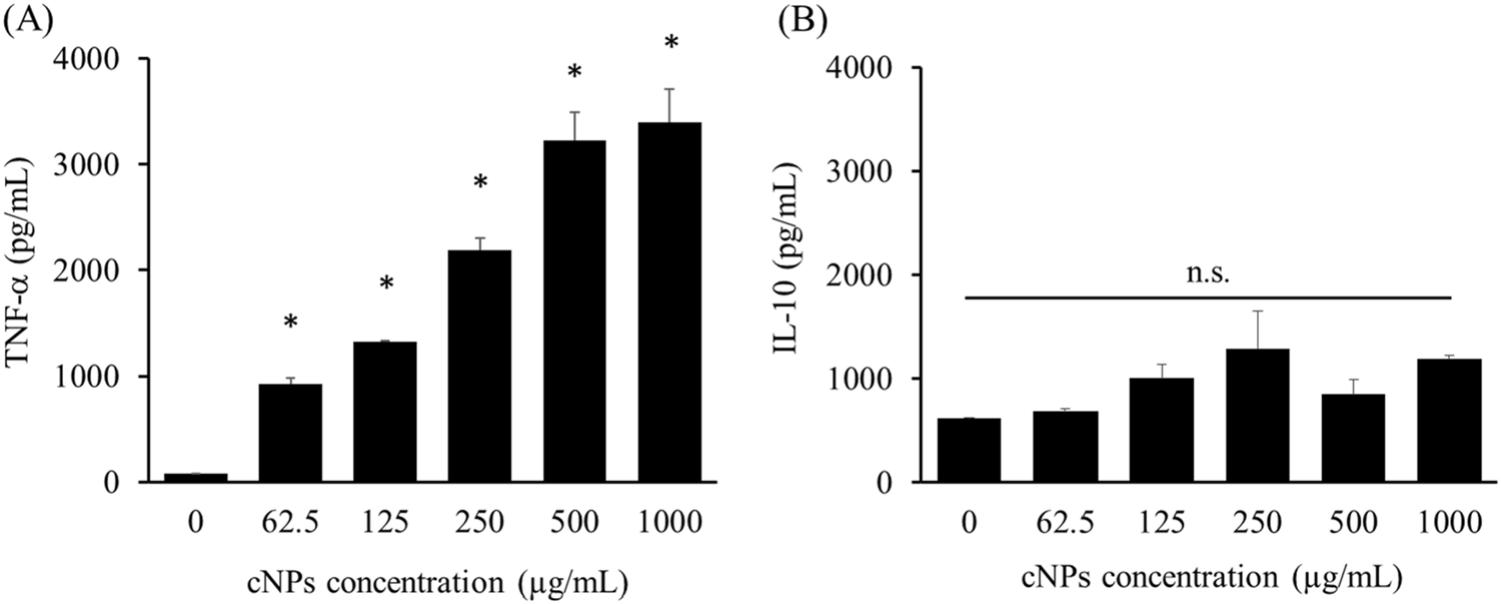
Cytokine release from RAW264.7 cells. **(A)** TNF-α and **(B)** IL-10 production from RAW264.7 cells after cNP addition. The supernatants of RAW264.7 cells after 24 h of cNP addition were collected for ELISA. Results are expressed as the mean ± SD of three samples. *p < 0.05 vs. 0 μg/mL cNP group (the control group). *n.s.* not significant.

The immunological activity of cNPs, being a xenobiotic product, was determined by examining cytokine release from RAW264.7 cells after addition of cNPs. RAW264.7 cells significantly released the proinflammatory cytokine tumor necrosis factor (TNF)-α after incubation with cNPs (Fig. 5A), though no significant release of anti-inflammatory cytokine interleukin (IL)-10 was detected (Fig. 5B).

### Combined effect of cNPs and RAW264.7 cells on the proliferation of colon26/fluc cells

**Figure 6:**
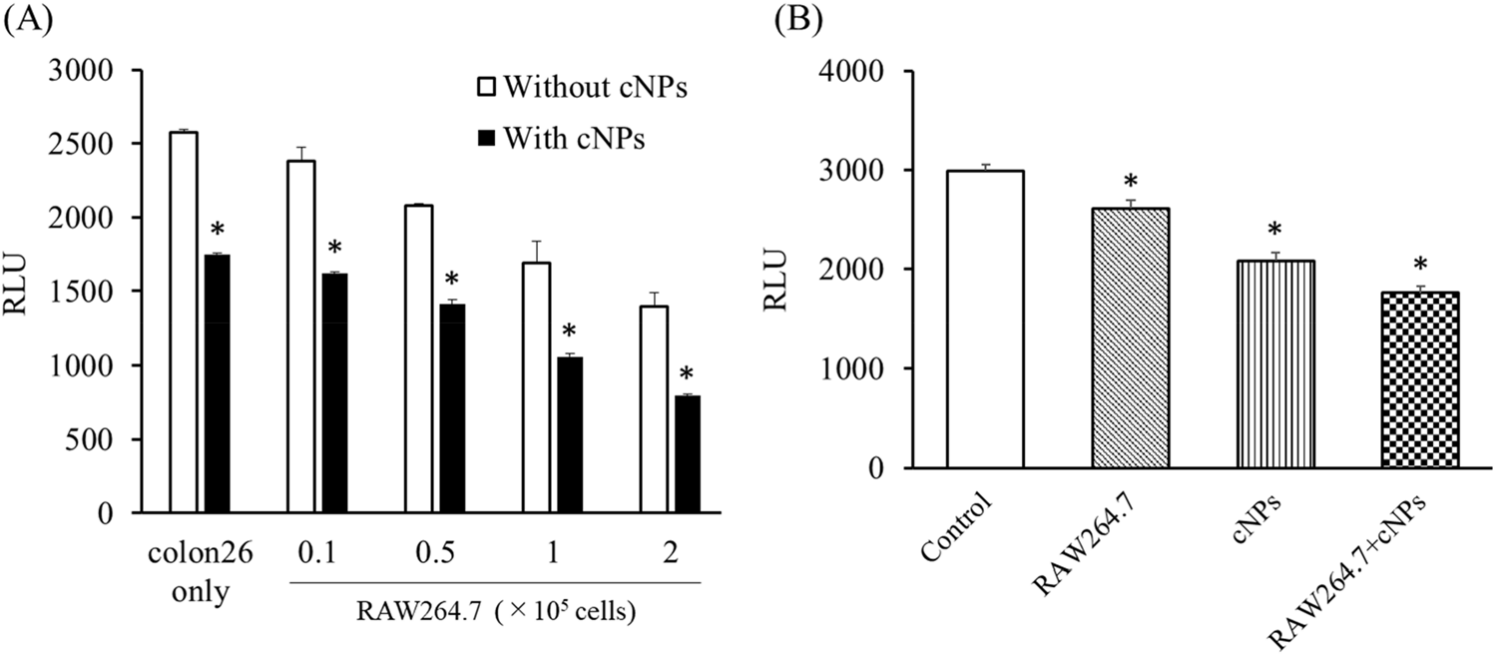
Combined effect of cNPs and RAW264.7 cells on the proliferation of colon26/fluc cells. **(A)** Co-incubation of RAW264.7 cells with colon26/fluc cells with or without cNPs. After 24 h-incubation, cells were lysed, and luciferase activity was measured. Results are expressed as the mean ± SD of three samples. *p < 0.05 vs. without cNP (no treatment) group. **(B)** Colon26/fluc cells were seeded onto the bottom of Transwell, and RAW264.7 cells with or without cNPs were added to the insert well (upper bottom). After 24 h-incubation, colon26/fluc cells were lysed and luciferase activity was measured. Results are expressed as the mean ± SD of three samples. *p < 0.05 vs. control group.

To examine whether the cNP-induced responses in RAW264.7 cells affect the proliferation of tumor cells, the number of firefly luciferase (fluc) stably expressing-colon26 (colon26/fluc) cells was evaluated after co-culture with RAW264.7 cells and cNP treatment (Fig. 6A). The number of colon26/fluc cells decreased when co-cultured with RAW264.7 cells in a cell number-dependent manner. In addition, the cell number was further reduced by adding cNPs. Moreover, colon26/fluc cells and RAW264.7 cells were separately cultured using Transwell culture plates (Fig. 6B). The findings demonstrated that RAW264.7 cells or cNPs reduced the number of colon26/fluc cells, and their combination further reduced the number. These results indicated that cNPs suppressed the proliferation of colon26/fluc cells by direct and indirect mechanisms through activation of immune cells.

### Antitumor effect of cNPs in tumor-bearing mice

**Figure 7:**
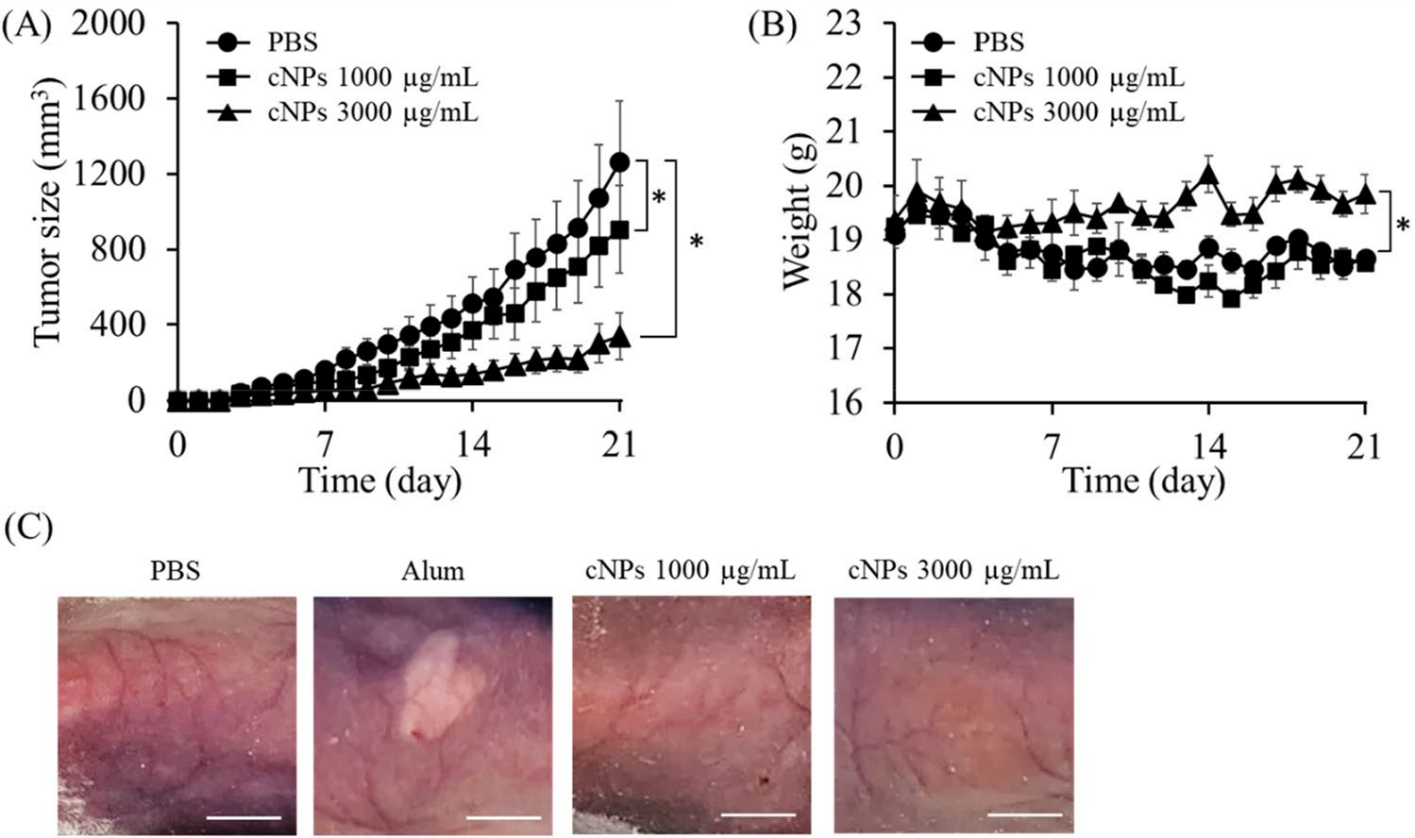
Tumor growth suppression in tumor-bearing mice after cNP administration. **(A)** Tumor size in tumor-bearing mice. Colon26 cells were subcutaneously injected into the back area of BALB/c mice. After the tumor size reached 50 mm^3^, cNPs at concentrations of 1000 or 3000 μg/mL (50 μL) were intratumorally administered every day. Results are expressed as the mean ± SD of three mice. *p < 0.05 vs. PBS group. **(B)** Body weight changes in different treatment groups. The change of body weight in tumor-bearing mice after cNP treatment. Results are expressed as the mean ± SD of three mice. *p < 0.05 vs. PBS group. **(C)** Skin inflammation in mice after cNP administration. 1000 or 3000 μg/mL cNPs, alum, and PBS (50 μL) were subcutaneously administered into the back area of BALB/c mice. After 24 h, the skin was observed. Scale bars indicate 5 mm.

Further, we examined the effect of cNPs on tumor growth in tumor-bearing mice. Figure 7A shows the tumor size against time in colon26 tumor-bearing mice. cNPs significantly suppressed tumor growth in a dose-dependent manner. The tumor volume was approximately 910 mm^3^ and 340 mm^3^ at cNP concentrations of 1000 μg/mL and 3000 μg/mL, respectively, which were significantly smaller than that in the control group (approximately 1260 mm^3^). Body weight tended to decrease in the control and 1000 μg/mL cNP groups, probably due to tumor burden; however, the weight was maintained in 3000 μg/mL cNP group (Fig. 7B). Figure 7C shows the skin surface of the injection site after subcutaneous injection of PBS, Imject Alum Adjuvant (alum), or cNPs. Alum was used as a positive control to induce inflammation, and therefore the group showed erythema and edema at the injection site, however, these effects were not observed in cNP groups.

### Evaluation of organ toxicity after cNP injection

To evaluate whether cNPs induced toxicity in mice, we performed hematoxylin and eosin (H&E) staining of organ sections and measured the aspartate aminotransferase (AST), alanine aminotransferase (ALT), and creatinine (Cre) levels in the serum of mice at 7 days after daily subcutaneous injections of cNPs at a dose of 1000 µg/mL. The H&E-stained sections of the cNP group showed no significant changes, indicating that cNPs induced little toxicity in these organs (Fig. S3A). In addition, the ALT, AST, and Cre levels in the serum were only minimally increased by cNP injections (Fig. S3B–D).

## Discussion

Human cell-derived EVs and synthetically prepared NPs, including liposomes, have potential application as drug delivery carriers that can improve the therapeutic index of various biologically active compounds. However, these carriers are lacking one or more properties required for such applications, including ease of preparation, low cost, and large-scale and reproducible production. Notably, cNPs prepared in the present study satisfied these requirements, as they were prepared on a large scale (Table 1) from inexpensive materials using a simple preparation process (Fig. 1A), and had a uniform size that was similar to liposomes (Fig. 1B, Table 1). cNPs contained LPCs, PCs, LPEs, PEs, PGs, PSs and SMs, and these phospholipids were concentrated in cNPs compared to those in corn homogenized juice (Table S1). When we evaluated the shape of cNPs by TEM, we revealed that cNPs are nanoparticles with a hollow structure (Fig. 1C, Fig. S1), but it is not known why they have such a structure. It is necessary to clarify it in the future studies. The yield of PC-Lips was approximately 3.2 × 1.1 ± 10^11^ NPs/mL by employing the commonly used thin film hydration method. In contrast, the yield of cNPs was approximately 36 × 2 ± 10^11^, which was around tenfold higher than that of the PC-Lips (Table 1), indicating that cNPs can be mass-produced. Thus, our findings suggest that cNPs are nanoparticles that can be mass-produced using a simple protocol and are good candidates for drug delivery.

In general, NPs are taken up by cells via endocytosis; the pathways can be broadly categorized as clathrin-dependent endocytosis occurring in the non-lipid raft membrane domain, and phagocytosis and micropinocytosis occurring in the mixed membrane domains, depending on the cell membrane domains involved. Caveolae-dependent and cholesterol-dependent endocytosis pathways occur in the lipid raft domain^31,32^. The uptake pathways of epNPs have been evaluated in previous studies. For example, the uptake of ginger-derived NPs by colon26 cells was not inhibited by CPZ or amiloride^13^, whereas the uptake of grapefruit-derived NPs by RAW264.7 cells was significantly inhibited by CPZ^33^. The results of the present study demonstrated that cNPs were preferentially taken up by colon26 cells compared to noncancerous cells (Fig. 2A,B). Since MβCD is an inhibitor of cholesterol-dependent endocytosis pathway at the lipid raft domain, this pathway might be partly involved in the uptake of cNPs by colon26 cells (Fig. 2C,D). It has been reported that cNPs have a lipid bilayer membrane structure (Fig. 1C), though not studied in detail to date with respect to lipid composition. A previous report showed that the lipid composition of the edible part of corn, which was used for the preparation of cNPs, is 52.8% triglycerides, 18.4% glycolipids, and 28.8% phospholipids^34^. Considering this as the lipid composition of cNPs, it is suggested that the hydrophobic interaction of the lipid bilayer of cNPs with the lipid rafts on the cell surface could contribute to the uptake of cNPs. In addition, it has been reported that cancer cells contain higher levels of lipid rafts than their normal non-tumorigenic counterparts, which contribute to oncogenic signaling and promote tumor progression in cancer^35^, as evidenced by human breast cancer cell line MCF-7 having more lipid rafts than human breast epithelial cell line MCF-10A^36^. These findings provide sufficient basis for efficient uptake of cNPs by colon26 cells due to the presence of adequate lipid rafts.

The present study involved three types of cells to demonstrate biological activities of cNPs. The results showed that cNPs inhibited the proliferation of tumor cells more efficiently than that of non-cancerous cells (Fig. 3A,B). This effect may be related to the high proliferation rate of cancer cells and efficient cellular uptake of cNPs by tumor cells (Fig. 2B). Similar results were obtained for human cancer cell lines (Fig. S2), indicating that cNPs have anti-proliferative activity against both mouse and human cancer cells. NPs derived from plants other than corn have also been reported to exhibit biological activities. For example, various plant sap-derived NPs have been reported to exhibit anti-proliferative activity of varying extent in various tumor cell lines^37^. Additionally, citrus limon-derived NPs were reported to induce TNF-related apoptosis-inducing ligand-mediated cell death^38^. High proliferation rate of cancer cells is related to the accelerated expression of proliferation-related proteins^39^. A recent study reported that survivin, a cell proliferation-related protein, was expressed in colon cancer cells but not in normal colon^40^, and siRNA targeting survivin exhibited a great anti-proliferative activity^41^. These studies are suggestive of the contribution of differences in the expression levels of proliferation-related proteins in anti-proliferative activity of cNPs.

epNPs have been reported to contain various biologically active molecules^13,14,16,17^. Lutein and zeaxanthin, the xanthophylls present in corn^28,29^, are known to exhibit antitumor effects through regulation of cell cycle or cell apoptosis^42–44^. It has been reported that zeaxanthin induced cell cycle arrest at the G2/M phase and apoptosis through the ROS-mediated MAPK, AKT, NF-κB, and STAT3 signaling pathways^43^. High-performance liquid chromatography analysis in the present study confirmed the presence of lutein and zeaxanthin in cNPs (data not shown). Our results are consistent with these studies because addition of cNPs resulted in cell cycle arrest and cell death in colon26 cells (Fig. 4), indicating that xanthophylls could be responsible for biological activities of cNPs, including anti-tumor activity. In addition, recent studies have reported that microRNAs are upregulated in plants in response to growth process or external stimuli^45^, including corn^27^. Interestingly, microRNAs, including miR156 and miR396, have been detected in the serum of corn-fed pigs^46^. These exogenous microRNAs may regulate “cross-kingdom” gene expression in other species, and plant-derived NPs containing these exogenous microRNAs are thought to play an important role in gene regulation. In recent studies, plant-derived microRNAs, such as miR156, miR159, and miR167, suppressed the proliferation of enterocytes or breast cancer^47–49^. Thus, microRNAs encapsulated in cNPs have potential to induce cell cycle arrest in cancer cells. Further studies are needed to confirm the relationship between anti-tumor activity and biologically active molecules in cNPs.

Immune cells, including RAW264.7 cells, release cytokines upon stimulation with pathogen-associated molecular patterns (PAMPs) or Toll-like receptor (TLR) agonists. For example, TLR9 agonists such as CpG oligodeoxynucleotides were recognized by TLR9, followed by the release of IL-6 and TNF-α from TLR9-positive cells^50^. Cytokines play important roles in host defense and homeostasis, and several attempts have been made to treat various diseases with cytokines^51,52^. There have been several reports on epNP-induced cytokine production^13,14,16,53,54^. For example, ginger- and ginseng-derived NPs induced IL-10 production in RAW264.7 cells^13^, and TNF-α and IL-6 production in macrophage cells^54^. In the present study, we demonstrated that cNPs induced the release of TNF-α from RAW264.7 cells (Fig. 5). However, the detailed mechanism of cytokine production by cNPs was not known in this study. Previous studies reported that TLR4 recognized ginseng-derived NPs as PAMPs, thereby affecting the cascade of the TLR/myeloid differentiation factor 88-dependent signaling pathway, followed by the release of TNF-α in macrophages^54^. These findings are suggestive of a similar mechanism for the release of TNF-α following cNP addition, however, the detail mechanism needs to be examined. In addition, ginseng-derived NPs polarized tumor-associated macrophages to the M1 type and exhibited antitumor effect through TNF-α production^55^ in the tumor. In the present study, cNPs exerted indirect antitumor effects by activating RAW264.7 cells (Fig. 6). Several types of immune cells, including tumor-associated macrophages (TAMs), are present in tumor tissues. Administered cNPs can be taken up by TAMs, which then release TNF-α and other antitumor cytokines. Therefore, cNP-induced immune activation may be useful for treating cancer.

Anti-tumor activity of materials in tumor-bearing mice can be evaluated through directly injecting them intratumorally. Using a colon26 tumor-bearing xenograft mouse model, we demonstrated that cNPs could significantly suppress tumor growth with minimal adverse effects (Fig. 7). No significant adverse effects were detected after daily subcutaneous injections of cNPs, suggesting that cNP are safe nanoparticles. The in vitro studies suggested that cNPs suppressed tumor growth in mice by inhibiting the proliferation of colon26 cells (direct effect) and through TNF-α production by activation of macrophages and other immune cells infiltrating into the tumor (indirect effect). These two effects could synergistically or additively contribute to the marked in vivo antitumor effect of cNPs.

## Methods

### Materials

Fetal bovine serum (FBS) was obtained from Biosera (East Sussex, UK); nonessential amino acid solution (NEAA), CPZ, sodium phosphotungstate, and 4% PFA were obtained from Nacalai Tesque, Inc. (Kyoto, Japan). PC, cholesterol, and PE were kindly provided by Nippon Fine Chemical Co., Ltd. (Tokyo, Japan). Chloroform, methanol, penicillin–streptomycin-L-glutamine solution (× 100) (PSG), and PI were obtained from Wako Pure Chemical Industries, Ltd. (Osaka, Japan). MβCD was obtained from Tokyo Chemical Industry Co., Ltd. (Tokyo, Japan). EIPA was obtained from R&D Systems, Inc. (Minneapolis, MN, USA). Filipin III was obtained from Santa Cruz Biotechnology, Inc. (Santa Cruz, CA, USA). Alum was obtained from Thermo Fisher Scientific (Waltham, MA, USA). Dulbecco’s modified Eagle’s medium (DMEM) and Roswell Park Memorial Institute (RPMI) 1640 medium were obtained from Nissui Pharmaceutical Co., Ltd. (Tokyo, Japan). All other chemicals used were of the highest commercially available grade.

### Animals

Eight-week-old BALB/c female mice were purchased from Sankyo Labo Service Co., Inc. (Tokyo, Japan) and maintained under specific pathogen-free conditions. The protocols for experiments involving animals were approved by the Institutional Animal Experimentation Committee of the Tokyo University of Science. All experiments involving animals were conducted in accordance with the principles and procedures outlined in the National Institutes of Health Guide for the Care and Use of Laboratory Animals and the ARRIVE guidelines.

### Cell culture

RAW264.7, colon26, and colon26/fluc cells^56^ were obtained from Professor Yoshinobu Takakura (Department of Biopharmaceutics and Drug Metabolism, Graduate School of Pharmaceutical Sciences, Kyoto University, Kyoto, Japan), and cultured in RPMI medium supplemented with 10% heat-inactivated FBS and PSG at 37 °C in humidified air containing 5% CO_2_. NIH3T3 cells (RCB2767) were provided by the Riken BRC through the National BioResource Project of the MEXT/AMED (Ibaraki, Japan). MDA-MB-231 cells were provided by Professor Fumio Fukai (Tokyo University of Science) and Panc-1 cells were provided by Professor Mutsunori Murahashi (Jikei University School of Medicine, Tokyo, Japan). These cells were cultured in DMEM supplemented with 10% heat-inactivated FBS and PSG at 37 °C in humidified air containing 5% CO_2_.

### Preparation of PC liposomes (PC-Lips)

PC, cholesterol, and phosphatidylethanolamine were mixed at a molar ratio of 10:5:1 and dissolved in 2 mL chloroform in a round-bottom flask. A lipid film was formed on the wall surface of the flask by solvent evaporation under reduced pressure using a vacuum pump in a water bath. Then, 1 mL PBS was added, and crude PC liposomes were prepared by sonication at 70 °C for 2 min using an ultrasonic cleaner (Sono Cleaner, Kaijo, Tokyo, Japan)^57^. They were extruded 5 times through a Whatman Nuclepore Track-Etched Membrane with a 100-nm pore size (Cytiva, Tokyo, Japan) at 70 °C. After centrifugation at 10,000 × *g* for 10 min to remove aggregates, PC-Lips were obtained from the supernatant. PC-Lips were used as control NPs for cNPs because they share similar characteristics such as the particle size with cNPs.

### Preparation of cNPs

Super sweet corn was purchased from a local fresh market and washed thoroughly with distilled water. The edible portion of corn (100 g) was vigorously homogenized with 100 mL of distilled water using a food processor for 2 min. The homogenized juice was sequentially centrifuged at 2000 × *g* for 20 min, 5000 × *g* for 30 min, and 10,000 × *g* for 1 h at 4 °C. The supernatant was filtered through a 0.45 μm-pore size syringe filter (Minisart NML, Sartorius, Göttingen, Germany) to exclude rough residues. Approximately 38 mL of the filtered sample was added to 2 mL of 60% sucrose solution in a tube, and then ultra-centrifuged at 100,000 × *g* for 120 min at 4 °C using Optima XL-K with an SW28 rotor (Beckman Coulter, Inc., Brea, CA, USA). The supernatant was carefully removed from the top, and the fraction (approximately 2 mL) containing yellow-colored cNPs that was present just above the 60% sucrose solution was collected carefully without disturbing the sucrose solution.

### Characterization of cNPs

The particle size and zeta potential of cNPs were measured by DLS using an ELSZ-2000ZS instrument (Otsuka Electronics Co., Ltd, Osaka, Japan). The particle number was measured using a ZetaView Laser Light Scattering Microscope (Particle Matrix GmbH, Microtrac, Meerbusch, Germany). For TEM imaging, a drop of cNPs was deposited onto the surface of a carbon-coated copper grid and negatively stained with 1% sodium phosphotungstate for 3 min, and the sample was dried at room temperature (approximately 22 °C). Then, the sample was observed using an H-7650 TEM (Hitachi High-Tech Co., Ltd, Tokyo, Japan) operated at 100 kV. The protein concentration of cNPs was measured by the Bradford assay^58^, and used to standardize the concentration of cNPs.

### Phospholipid analysis by LC–MS/MS

To analyze the phospholipid components in cNPs, the LC–MS/MS Method Package for Phospholipid Profiling (Shimadzu Co., Kyoto, Japan) was used according to the manufacturer's instructions. The library of phospholipid targets in the method package includes PC, PE, PG, PI, PS, and SM. Briefly, cNPs and corn homogenized juice prepared at a concentration of 100 µg protein/mL were diluted tenfold with methanol containing 0.1% formic acid for mass spectral analysis. These sample solutions (5 μL) were injected into a Kinetex C8 column (2.1 mm I.D. × 150 mm., 2.6 µm, Phenomenex, Torrance, CA, USA) at a flow rate of 0.5 mL/min. The samples were eluted by a gradient of mobile phase A (20 mM ammonium formate in water) and mobile phase B (isopropanol:acetonitrile = 1:1 v/v). The concentration of the mobile phase B was programmed as 20% (0 min)–20% (1 min)–40% (2 min)–92.5% (25 min)–92.5% (26 min)–100% (35 min)–20% (38 min). The oven temperature was set at 45 °C. Data processing and lipid identification/quantification were performed using LabSolutions software (version 5.99 SP2; Shimadzu Co.). The analytical results were obtained from multiple reaction monitoring transitions and generally used for lipid analysis^59–61^. The peak area ratio was calculated by dividing the area of the sample peak by the area of the internal standard (IS) peak. As IS, 17:0–20:4 PI (Avanti Polar Lipids, Alabaster, AL, USA) was added to each sample at a final concentration of 0.38 µmol/L. Only phospholipids with a peak area ≥ 5000 were analyzed.

### Fluorescent labeling of NPs

cNPs were labeled with a fluorescent lipophilic dye, DiI (Thermo Fisher Scientific). In brief, 0.1 mg/mL DiI solution (10 μL) was added to 1000 μg/mL cNPs (1 mL), and the mixture was incubated for 30 min at 37 °C. DiI-labeled cNPs were then purified using 100-kDa ultrafiltration (Amicon Ultra-15, Merck KGaA, Darmstadt, Germany). DiI-labeled PC-Lips were prepared in a similar manner.

### Cellular uptake of DiI-labeled cNPs

Colon26, NIH3T3, and RAW264.7 cells were seeded in 4-well chambered cover glass (IWAKI; AGC Techno Glass Co., Ltd., Chiba, Japan) at a density of 1 × 10^5^ cells/well and cultured overnight at 37 °C. The medium was then replaced with fresh culture medium containing 350 μg/mL DiI-labeled cNPs. After 3 h of incubation, the cells were fixed with 4% PFA for 30 min on ice, and the cells were washed three times with PBS. Subsequently, Vectashield Antifade Mounting Medium with DAPI (Vector Laboratories Inc., Burlingame, CA, USA) was added. The cells were imaged using a Leica SP8 laser scanning confocal microscope (Leica, Wetzlar, Germany) using the LAS X Life Science software. For flow cytometric analysis, the cells fixed with 4% PFA were collected using a cell scraper, and then filtered through a 70 μm cell strainer (Becton Dickinson). Cellular uptake of DiI-cNPs or PC-Lips was quantitatively analyzed using a BD FACSCalibur flow cytometer (Becton Dickinson, San Jose, CA, USA) and FlowJo software ver8.7 (Becton Dickinson).

### Elucidation of cellular uptake pathway of cNPs

To elucidate the cellular uptake pathways of cNPs, endocytosis inhibitors (10 mM MβCD, 30 μM CPZ, 40 μM EIPA, and 0.5 μg/mL Filipin III) were added to colon26 cells and incubated for 40 min at 37 °C. DiI-labeled cNPs were then added to the cells and incubated for 3 h at 37 °C. The cells were fixed on a glass chamber slide using 4% PFA for fluorescence imaging under a confocal microscope. Cellular uptake of DiI-cNPs was quantitatively analyzed using a BD FACSCalibur flow cytometer and FlowJo software ver8.7, as described above.

### Cell number and proliferation assay

Colon26, NIH3T3, and RAW264.7 cells were seeded in 96-well culture plate at a density of 5 × 10^3^ cells/well and incubated for 24 h at 37 °C. Culture medium was then replaced with fresh medium containing various concentrations of cNPs. After 24 h of incubation, cell number was measured using the CCK8 (Dojindo Laboratories, Kumamoto, Japan). The experiment was repeated except the medium was replaced with fresh medium containing 1000 μg/mL cNPs. The cell number was measured using the CCK8 after 6, 12, 24, and 48 h of incubation.

### Cell cycle analysis

Colon26 cells were seeded in six-well culture plate at a density of 1 × 10^5^ cells and incubated for 24 h. The cells were washed with PBS, and cNPs at concentrations of 250, 500, and 1000 μg/mL were added to them. After 24 h incubation, the cells were washed with PBS and fixed with 70% ethanol at 4 °C for 3 h. PI (50 μg/mL) was added to the cells, which were then incubated for 30 min at 4 °C in the dark. The cells were analyzed using a BD FACSCalibur flow cytometer, and cell cycle analysis was performed using the FlowJo software version 8.7.

### Analysis of dead cells

Colon26 cells were seeded into a 6-well culture plate at a density of 1 × 10^5^ cells and incubated for 24 h. The cells were washed with PBS, and cNPs were added at a concentration of 1000 μg/mL. The cells were collected using a cell scraper after 1, 3, and 6 h of incubation. Immediately, PI solution (50 μg/mL) was added to the cells, which were then incubated for 5 min at 4 °C in the dark. The cells were analyzed using a BD FACSCalibur flow cytometer, and the proportion of dead cells was calculated using the FlowJo software version 8.7.

### Cytokine measurement by ELISA

RAW264.7 cells were seeded in 96-well culture plate at a density of 1 × 10^4^ cells/well and incubated for 24 h. The medium was replaced with fresh medium with or without cNPs at various concentrations. After 24 h incubation, the concentration of TNF-α and IL-10 in the supernatant were measured using Mouse TNF-α ELISA MAX Deluxe Set (BioLegend, San Diego, CA, USA) and Mouse IL-10 Uncoated ELISA kit (Thermo Fisher Scientific, Waltham, MA, USA), respectively.

### Effect of cNPs and RAW264.7 cells on the proliferation of colon26/fluc cells

Colon26/fluc cells were seeded in 12-well culture plate at a density of 1 × 10^5^ cells/well and incubated for 24 h. RAW264.7 cells (with varying numbers) and 1000 μg/mL cNPs were added to colon26/fluc cells. After 24 h incubation, cells were washed three times with PBS. Subsequently, the cells were lysed using 100 μL cell lysis buffer and incubated for 3 h on ice for complete lysis. Luciferase activity in cell lysates was measured using Picagene luciferase assay kit (Toyo B-Net Co., Ltd., Tokyo, Japan). In addition to this, colon26/fluc cells were seeded in the lower chamber of Transwell cell culture plates at a density of 1 × 10^5^ cells/well. After 24 h incubation, RAW264.7 cells (1 × 10^5^ in number) and cNPs at a concentration of 1000 μg/mL were added to the insert (upper chamber). After 24 h of incubation, colon26/fluc cells in the lower chamber were washed three times with PBS and lysed using 100 μL of cell lysis buffer. Luciferase activity in cell lysates was measured as described above.

### Anti-tumor effect of cNPs in a subcutaneous xenograft mouse model

To prepare a subcutaneous tumor xenograft mouse model, colon26 cells suspended in PBS (1 × 10^6^ cells/100 μL) were subcutaneously injected into the back area of BALB/c mice. When the tumor size reached 50 mm^3^, the mice were randomly assigned to three different treatment groups, and then saline or cNPs (50 or 150 μg/shot) was injected daily into the tumor for 21 days. Tumor size and body weight of the mice were measured at the time of the sample injection. Tumor volume was calculated as follows: volume = 1/2 LW^2^, where L is the long diameter and W is the short diameter of the tumor^62^. At the end point of the experiment, mice were euthanized under isoflurane anesthesia.

### Evaluation of adverse effects after cNP injection

The hair on the back of BALB/c mice was removed using a clipper, and then 50 μL of PBS (vehicle), alum (40 mg/mL aluminum hydroxide and 40 mg/mL magnesium hydroxide), or cNPs (1000 or 3000 μg/mL) was subcutaneously injected. The injection site was photographed after 24 h. After the experiment, the mice were euthanized under isoflurane anesthesia. To evaluate organ toxicity, PBS or cNPs (1000 μg/mL) were subcutaneously injected into the back of BALB/c mice every day. On day 7, the organs (heart, lung, liver, spleen, and kidney) and blood were collected, and the blood was incubated on ice overnight. The serum was collected by centrifugation at 2000 × *g* for 20 min. The extracted organs were fixed in 4% PFA and sectioned at 10-μm thickness using a cryostat for staining with H&E. The tissue sections were observed using a digital microscope (BZ-9000, Keyence, Osaka, Japan). Additionally, the AST, ALT, and Cre levels in the serum were measured using a transaminase CII-test Wako kit (Wako Pure Chemical Industries, Ltd., Osaka, Japan) and LabAssay Creatinine kit (Wako Pure Chemical Industries, Ltd.), respectively.

### Statistical analysis

Statistical differences were evaluated by one-way analysis of variance (ANOVA) followed by the Dunnett’s test for multiple comparisons or Student’s t-test for comparison between two groups. Statistical significance was set at p < 0.05.

## Supplementary Information

Supplementary Information.
