# Supplementary material for: Development of nanoparticles derived from corn as mass producible bionanoparticles with anticancer activity
Source: Sci Rep. 2021 Nov 24;11:22818. doi: 10.1038/s41598-021-02241-y (PMC8613273; doi:10.1038/s41598-021-02241-y)
Supplement: Supplementary file 1 — Supplementary Information. [file 41598_2021_2241_MOESM1_ESM.pdf]

Supplementary Information

Development of nanoparticles derived from corn as mass producible bionanoparticles with anticancer activity

Daisuke Sasaki[1], Kosuke Kusamori[1], Yukiya Takayama[1], Shoko Itakura[2], Hiroaki Todo[2], and Makiya Nishikawa[1*]

[1]Laboratory of Biopharmaceutics, Faculty of Pharmaceutical Sciences, Tokyo University of Science, 2641 Yamazaki, Noda, Chiba 278-8510, Japan

[2]Faculty of Pharmacy and Pharmaceutical Sciences, Josai University, 1-1 Keyakidai, Sakado, Saitama 350-0295, Japan

*Corresponding author: Makiya Nishikawa, Ph.D.

Tel.: +81-4-7121-4450

Fax.: +81-4-7121-4450

E-mail address: makiya@rs.tus.ac.jp

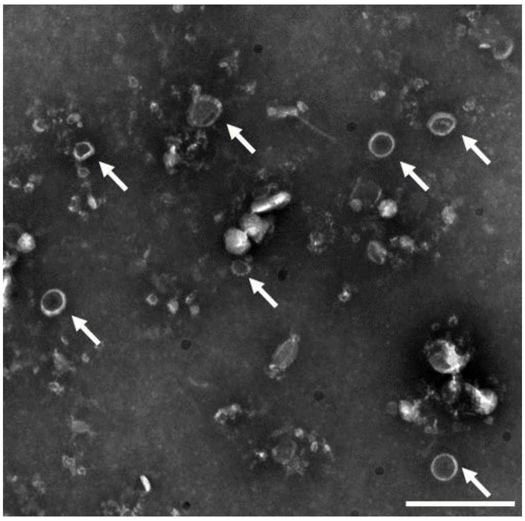

Fig. S1. TEM image of cNPs at low magnification. A TEM image was captured using H-7650 TEM. The scale bar indicates 500 nm. White arrows indicate cNPs.

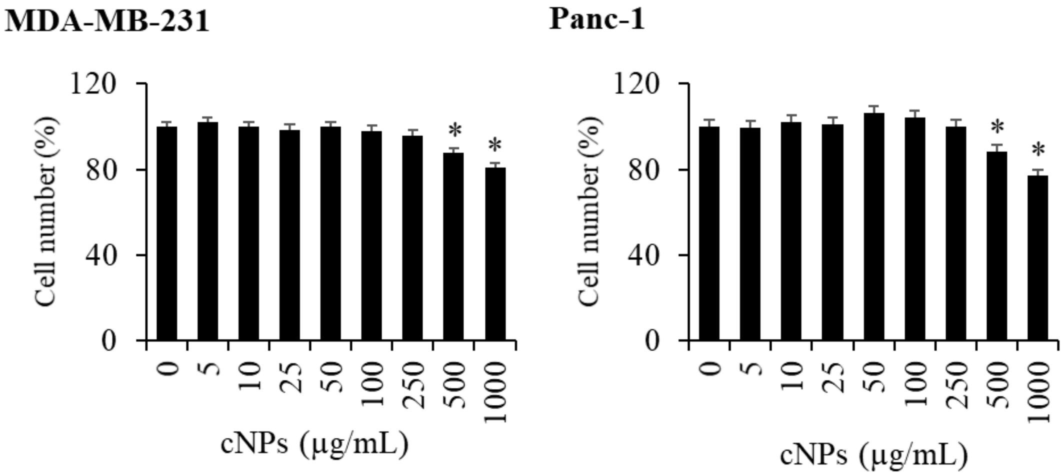

Fig. S2. Cell number after incubation with cNPs. Cell number was measured by CCK8 assay after 24 h of cNP addition. MDA-MB-231 and Panc-1 cells were incubated with 5–1000 μg/mL of cNPs. Results are expressed as the mean ± SD of four samples. *p < 0.05 vs. 0 μg/mL cNP

group.

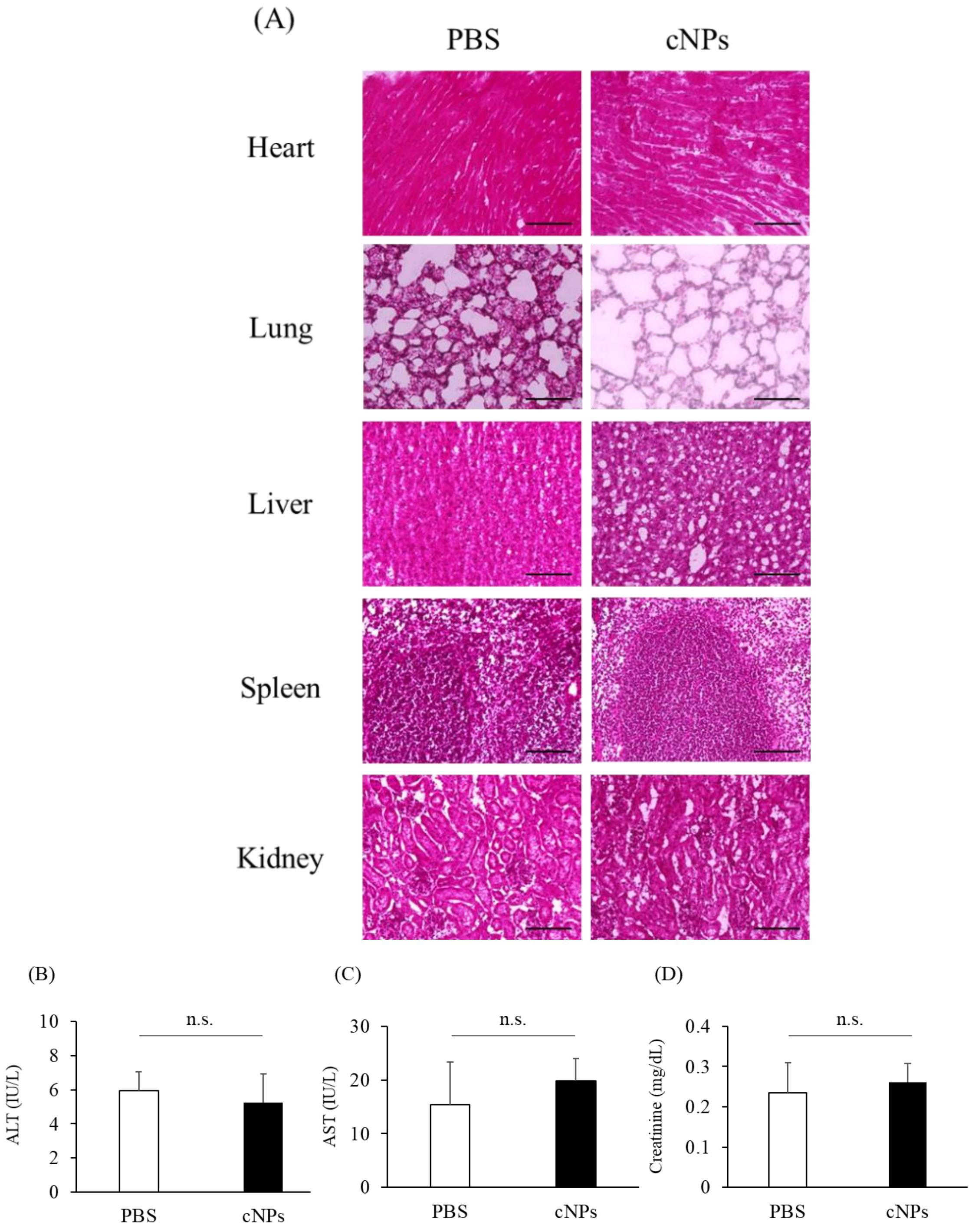

Fig. S3. Adverse effects in mice after cNP injection. PBS or cNPs (1000 μg/mL) were

subcutaneously injected to the back area of BALB/c mice every day. On day 7, organs and blood were collected, and blood was incubated on ice overnight. The extracted organs were sectioned, and H&E stained. (A) H&E stained tissue sections. Scale bars indicate 100 μm. (B-D) Serum levels of AST (B), ALT (C), and creatinine (D).

Table S1. Peak area ratio of phospholipids in cNPs

Phospho-lipids	Acyl chain*	Peak area ratio (sample/IS)	
		cNPs	Corn homogenized juice (before centrifugation)
LPC	16:0	0.224 ± 0.032	0.070 ± 0.003
	18:1	0.107 ± 0.016	0.022 ± 0.003
	18:2	0.115 ± 0.020	0.018 ± 0.002
PC	16:0-16:0	0.018 ± 0.005	N.D.
	16:0-18:0	0.020 ± 0.006	N.D.
	16:0-18:1	0.410 ± 0.049	0.142 ± 0.010
	16:0-18:2	0.597 ± 0.100	0.187 ± 0.012
	16:0-18:3	0.011 ± 0.003	N.D.
	18:0-18:1	0.011 ± 0.002	N.D.
	18:1-18:1	0.953 ± 0.137	0.297 ± 0.008
	18:1-18:2	1.303 ± 0.243	0.474 ± 0.007
	18:1-18:3	0.012 ± 0.006	N.D.
	18:2-18:2	1.010 ± 0.111	0.261 ± 0.025
	18:2-18:3	0.015 ± 0.005	N.D.
LPE	16:0	0.011 ± 0.003	N.D.
	18:1	0.016 ± 0.003	N.D.
PE	16:0-18:1	0.035 ± 0.007	0.006 ± 0.005
	16:0-18:2	0.100 ± 0.012	0.030 ± 0.007
	16:0-20:1	0.021 ± 0.003	N.D.
	18:1-18:1	0.076 ± 0.027	0.018 ± 0.006
	18:1-18:2	0.267 ± 0.046	0.085 ± 0.011
	18:2-18:2	0.440 ± 0.047	0.125 ± 0.012

PG	16:0-16:0	0.604 ± 0.017	N.D.
	14:0-20:1	0.009 ± 0.003	N.D.
	16:0-18:1	1.373 ± 0.168	0.036 ± 0.003
	16:0-18:2	0.139 ± 0.020	N.D.
PS	16:0-18:2	0.015 ± 0.006	N.D.
SM	18:1-20:0	0.020 ± 0.005	N.D.
	18:1-22:1	0.010 ± 0.005	N.D.

Results are expressed as the mean ± SD. N.D.: Not detected.

LPC: Lysophosphatidylcholine, PC: Phosphatidylcholine, LPE: Lysophosphatidylethanolamine, PE: Phosphatidylethanolamines, PG: Phosphatidylglycerol, PS: Phosphatidylserine, SM: Sphingomyelin.

*The numbers indicate the carbon number and degree of unsaturation of the acyl chains of phospholipids.
